# *MDR1* polymorphisms are associated with inflammatory bowel disease in a cohort of Croatian IBD patients

**DOI:** 10.1186/1471-230X-13-57

**Published:** 2013-03-27

**Authors:** Marko Brinar, Silvija Cukovic-Cavka, Nada Bozina, Katja Grubelic Ravic, Pave Markos, Agata Ladic, Marijana Cota, Zeljko Krznaric, Boris Vucelic

**Affiliations:** 1 Division of Gastroenterology and Hepatology, University Hospital Centre Zagreb, Zagreb, 10000, Croatia; 2 Clinical Institute for Laboratory Diagnosis, University Hospital Centre Zagreb, Zagreb, 10000, Croatia; 3 Nuvisan, Zagreb, Croatia

**Keywords:** *MDR1*, Crohn's disease, Ulcerative colitis, IBD

## Abstract

**Background:**

Inflammatory bowel diseases (IBD) are chronic diseases of unknown etiology and pathogenesis in which genetic factors contribute to development of disease. *MDR1*/*ABCB1* is an interesting candidate gene for IBD. The role of two single nucleotide polymorphisms, C3435T and G2677T remains unclear due to contradictory results of current studies. Thus, the aims of this research were to investigate the association of *MDR1* polymorphisms, C3435T and G2677T, and IBD.

**Methods:**

A total of 310 IBD patients, 199 Crohn's disease (CD) patients and 109 ulcerative colitis (UC) patients, and 120 healthy controls were included in the study. All subjects were genotyped for G2677T/A and C3435T polymorphism using RT-PCR. In IBD patients, review of medical records was performed and patients were phenotyped according to the Montreal classification.

**Results:**

Significantly higher frequency of 2677T allele (p = 0.05; OR 1.46, 95% CI (1.0-2.14)) and of the 3435TT genotype was observed among UC patients compared to controls (p = 0.02; OR 2.12; 95% CI (1.11-4.03). Heterozygous carriers for C3435T were significantly less likely to have CD (p = 0.02; OR 0.58, 95% CI (0.36-0.91)). Haplotype analysis revealed that carriers of 3435T/2677T haplotype had a significantly higher risk of having UC (p = 0.02; OR 1.55; 95% CI (1.06-2.28)).

**Conclusion:**

*MDR1* polymorphisms are associated with both CD and UC with a stronger association with UC.

## Background

Inflammatory bowel disease (IBD) encompasses Crohn's disease (CD) and ulcerative colitis (UC). Both CD and UC are chronic inflammatory disorders of the gastrointestinal tract with a combined incidence of 2–20 per 100,000 in the developed world [1]. In recent years, considerable progress in the field of IBD genetics has led to identification of a number of genetic factors involved in the pathogenesis of the disease. However, the underlying pathogenesis of IBD remains unclear. It is presumed that both disorders result as a consequence of defective mucosal barrier and a dysregulated immune response to a host's microbiota in genetically susceptible individual. In that respect, the multidrug resistance 1 (*MDR1*) gene, also known as adenosine triphosphate-binding cassette superfamily member 1 (*ABCB1*) is an interesting candidate gene for IBD for several reasons. *MDR1* encodes a transmembrane protein, P-glycoprotein 170 (Pgp) which functions as an ATP-dependent efflux transporter pump and is highly expressed in the epithelial surfaces of the intestine, biliary ductules, proximal tubules of kidneys and central nervous system [2-4]. Although its physiological substrate remains unknown, numerous, structurally different compounds, have been identified as a substrates of Pgp [5]. In the gut, Pgp is constitutively expressed on the apical surfaces of the superficial columnar epithelial cells in the intestine with levels of expression gradually rising from the duodenum to the distal parts of the intestine with the highest levels of expression in the distal small bowel and colon [6,7]. Although it physiological function in the gut remains unclear, such high levels of expression suggest not only a role in protection against xenobiotics but possibly a role in modulating host-bacterial interactions.

First clue to a possible role od *MDR1* in IBD pathogenesis comes from a mouse model in which *mdr1a* knock-out mice develop colitis resembling ulcerative colitis in a specific, pathogen free enviroment, that can be alleviated by a course of antibiotics [8]. Furthermore, *MDR1* is located within a region of suggestive IBD linkage on chromosome 7q [9]. Finally, *MDR1* gene expression is significantly reduced in the colonic tissue of UC patients [10]. These findings combined make *MDR1* an excellent both positional and functional IBD candidate gene.

Two single nucleotide polymorphisms (SNP), namely the triallelic G2677T/A (rs2032582) in exon 21 and C3435T (rs1045642) in exon 26, have been shown to correlate with Pgp expression [11-14]. Association of these SNP's with both CD and UC, either as a single marker or combined has been extensively studied. However, following initial positive associations subsequent studies have yielded contradictory results [15-30] (Table 1).

**Table 1:** **Summary of *MDR1 *association studies**

**Author**	**No of cases**	**Tested SNP**	**Population**	**Results**
Schwab et al.	149 UC	C3435T	German	T allele associated with UC (p = 0.049)
	126 CD			
Croucher et al.	307 UC	C3435T	German & British	No association
	562 CD			
Brant et al.	119 UC	C3435T G2677T	USA	G2677 associated with IBD (p = 0.003)
	409 CD			
Glas et al.	123 UC	C3435T	German	No association
	135 CD			
Potocnik et al.	144 UC	Multiple intron, exon and promoter polymorphisms	Slovenian	2677T associated with UC (p = 0.02); Risk and protective haplotypes identified for UC and CD
	139 CD			
Gazzouli et al.	85 UC	C3435T	Greece*	No association
	120 CD			
Ho et al.	335 UC	C3435T	Scottish	3435T associated with UC (p = 0.02); 3435T associated with extensive colitis (p = 0.003)
	268 CD	G2677T		
Onnie et al.	580 UC	C3435T	British	2677T associated with UC (p = 0.03)
	828 CD	G2677T		
Urcelay et al.	330 UC	C3435T	Spanish*	C3435T CC genotype associated with CD (p = 0.007)
	321 CD	G2677T		
Oostenbrug et al.	224 UC	C3435T	Dutch	No association
	533 CD			
Lal et al.	112 UC	C3435T	Canadian	3435T allele associated with CD (p = 0.02)
	247 CD			
Fiedler et al.	144 UC	C3435T	German	No association
	244 CD	G2677T		
Fischer et al.	149 UC	C3435T	Hungarian	No association
	265 CD	G2677T		
Ardizzone et al.	97 UC	C3435T	Italian	No association
	211 CD	G2677T		
Huebner et al.	401 UC	G2677T	New Zealand	G2677T protective for UC (p = 0.02)
	483 CD			

* Data not in Hardy-Weinberg equilibrium.

In summary, *MDR1* is an excellent positional and functional candidate gene for IBD. However, inconsistent results of previous studies make its role in IBD controversial. It has to be noted that a vast majority of association studies performed in Caucasians were done in North European populations known to have a higher incidence of IBD compared to regions of the Southern Europe suggesting possible genetic differences in these populations. Therefore, we aimed to investigate the association between *MDR1* G2677T/A and C3435T SNP and susceptibillity for UC and CD in an independent cohort of Croatian IBD patients and healthy controls.

## Methods

### Patients

A total of 199 CD patients and 109 UC patients followed up for IBD in the University Hospital Centre Zagreb from 01.01.2006 to 31.12.2010. were included. In all cases diagnosis of IBD was established according to standard clinical, radiological, endoscopic and histology criteria. Patients with unclassified IBD (IBDU) were excluded from the study. Upon inclusion, clinical records were reviewed for gender, age at diagnosis, localization and behavior of disease, extraintestinal manifestations and need for surgery. Localization and behavior of disease were defined according to Montreal classification [31]. The clinical characteristics of patients studied are summarized in Tables 2 and 3.

**Table 2:** Clinical characteristics of CD patients


Number of patients	199
Sex - no. of patients (%)	
Female	93 (46.7)
Male	106 (53.3)
Surgery – no of patients (%)	111 (56.3)
Age at diagnosis (years), median [interquartile range]	25.0 [18.50-31.10]
Age at surgery (years), median [interquartile range]	28.6 [22.80-36.80]
Disease duration to surgery (years), median [interquartile range]	3.0 [0.40-6.67]
Localization - no. of patients (%)	
Ileal ± UGI	64 (32.2)
Colon ± UGI	34 (17.1)
Ileocolon ± UGI	98 (49.2)
UGI	3 (1.5)
UGI any	29 (14.6)
Behaviour - no. of patients (%)	
Inflammatory ± perianal	89 (45.4)
Stricturing ± perianal	65 (33.2)
Penetrating ± perianal	42 (21.4)
Perianal (any)	70 (35.4)

**Table 3:** Clinical charateristics of UC patients


Number of patients	109
Sex - no. of patients (%)	
Female	57 (52.3)
Male	52 (47.7)
Surgery – no of patients (%)	21 (19.8)
Median [interquartile range] age at diagnosis (yrs)	29.9 [26.0-35.70]
Median [interquartile range] age at surgery (yrs)	37.3 [31.80-43.10]
Median [interquartile range] disease duration at surgery (yrs)	4.4 [1.0-7.75]
Localization - no. of patients (%)	
Proctitis	11 (10.2)
Left sided colitis	22 (20.4)
Pancolitis	75 (69.4)

A total of 120 age and sex-matched, unrelated, healthy volunteers recruited from the general population with no symptoms and no family history of IBD formed a control group. All volunteers were questioned regarding IBD symptoms as well as for symptoms of other immune mediated diseases such as rheumathologic conditions and psoriasis. Informed consent was obtained from all patients and controls prior to inclusion and the study was approved by the Ethics Committee of the University Hospital Centre Zagreb.

### Genotyping

Genomic DNA was extracted from peripheral lymphocytes using the salting out procedure [32]. *MDR1* genotypes of all patients were determined. For the *MDR1* exon 21 *G2677T*/*A*, and exon 26 *C3435T* variants, a Real-time PCR methods were performed using LightCycler (Roche Applied Sciences, Indianapolis, IN) with the Fast Start DNA Master plus HybProbe Master Mix [33-35]. To prevent genotyping errors DNA samples were genotyped in duplicate by RealTime PCR based method. Also every tenth DNA sample was checked by another method based on PCR-RFLP.

### Statistics

Basic group comparisons were performed using SPSS 17.0.1. (SPSS Inc., Chicago, IL) and Haploview 4.1. Differences in continuous variables were tested using the independent sample *t*-test and the Mann–Whitney *U* test for normally and not normally distributed variables, respectively.

Allele and genotype frequencies were compared between groups using the χ^2^ or Fischer’s exact test when appropriate. Two-sided p values along with odds ratios (OR) and 95% confidence intervals (CI) were calculated. Haplotype frequencies were estimated using the expectation-maximization (EM) algorithm [36] implemented in Haploview 4.1. Due to the low frequency of the A allele of the G2677T/A the carriers of that allele were excluded from further analysis. Significance was set at p < 0.05.

## Results

### Genotype analysis

Genotypes of both C3435T and G2677T/A were in Hardy-Weinberg equilibrium both in cases and in the control group. Distribution of allele and genotype frequencies for G2677T/A and C3435T SNP are listed in Table 4. We observed a trend toward higher frequency of the T allele of the C3435T SNP in UC patients compared to controls (53.7% vs. 44.6%; p = 0.051). In UC patients, a significantly higher frequency of the TT genotype was observed compared to controls (28.7% vs. 16.0%; p = 0.02; OR 2.12; 95% CI (1.11-4.03)) (Table 4). Marginally significant difference in the frequency of 2677T allele was observed in UC patients compared to controls (44.7% vs. 35.2%; p = 0.05; OR 1.46, 95% CI (1.0-2.14)). No significant differences in genotype frequencies for G2677T SNP were observed in UC cases compared to controls. No significant differences in allele frequencies of C3435T and G2677T/A were observed in CD cases compared to controls. However, association was detected on genotypic level with heterozygous carriers for C3435T having a significantly lower risk of having CD (p = 0.02; OR 0.58, 95% CI (0.36-0.91)). No significant differences in genotype frequencies for G2677T SNP were observed in CD cases compared to controls.

**Table 4:** ***MDR1 *C3435T and G2677T allele and genotype frequencies in CD**, **UC and control group**

	**Controls (n = 120)**	**CD (n = 199)**	**p value**	**UC (n = 109)**	**p value**
***C3435T***					
Allele frequencies (%)					
Allele C	132 (55.5)	208 (52.5)	0.47	100 (46.3)	0.051
Allele T	106 (44.5)	188 (47.5)		116 (53.7)	
Genotype frequencies (%)					
CC	32 (26.9)	61 (30.8)	0.52	23 (21.3)	0.35
CT	68 (57.1)	86 (43.4)	**0**.**02**	54 (50.0)	0.29
TT	19 (16.0)	51 (25.8)	0.05	31 (28.7)	**0**.**02**
***G2677T***/***A***					
Allele frequencies (%)					
Allele G	149 (64.2)	239 (61.6)	0.51	119 (55.1)	**0**.**05**
Allele T	83 (35.8)	149 (38.4)		97 (44.9)	
Genotype frequencies (%)					
GG	47 (40.5)	82 (42.3)	0.72	33 (30.5)	0.16
GT	55 (47.4)	75 (38.6)	0.16	53 (49.1)	0.69
TT	14 (12.1)	37 (19.1)	0.11	22 (20.4)	0.10

A high degree of linkage disequilibrium was observed between the two studied SNP's in our sample (D´ = 0.9). A significant difference in the frequency of carriers of 3435T/2677T haplotype between UC cases and controls was observed with carriers of that haplotype having a significantly higher risk of having UC (p = 0.02; OR 1.55; 95% CI (1.06-2.28)) (Table 5). No significant differences in 2-locus haplotype frequencies were observed in CD cases compared to controls.

**Table 5:** **Frequencies of two**-**locus *MDR1 *haplotypes in CD and UC cases and controls**

**Haplotype**	**Controls (%)**	**UC (n%)**	**p value**	**CD (%)**	**p value**
C3435/G2677	126 (53.4)	97 (44.6)	0.07	202 (51.0)	0.62
3435T/2677T	78 (33.1)	94 (43.2)	**0**.**02**	145 (36.6)	0.34
3435T/G2677	28 (11.9)	23 (10.5)	0.69	43 (10.9)	0.765
C3435/2677T	6 (2.6)	4 (1.7)	0.56	6 (1.5)	0.405

## Discussion

In this study, we investigated the association of *MDR1* polymorphisms and IBD in a cohort of well charaterized patients. We detected a significant association of C3435T and G2677T polymorphism and UC with higher frequency of mutant allele and/or genotype carriers in the UC patient group. In that respect, our results are in accordance with previously published results reporting the association of UC with G2677T and C3435T SNP [15,17,20,21,23]. Contrary to the previously published results, we detected no association of C3435T variant with pancolitis although a large proportion of our UC patient group had pancolitis (69.4%) [21]. Furthermore, due to the high LD between the two studied SNP's, we were able to investigate the association with two-locus haplotypes. Previous studies reported both *risk* and *protective* haplotypes in the same cohort suggesting a complex influence of *MDR1* in pathogenesis of disease [20,21]. We detected a significantly higher frequency of 3435T/2677T haplotype carriers in the UC patient group. The same haplotype is also a part of two haplotypes associated with UC in the study by Potocnik et al. performed on Slovenian IBD patients [20].

We found no significant difference in allele distributions of investigated SNP's between CD patient group and controls. However, we detected a significant association of C3435T heterozygous carriers with CD. Carriers of this genotype were significantly less represented in the CD patient group suggesting a protective effect. How exactly a heterozygous state confers protection is hard to explain at this moment. However, the same genotype was also found to be protective in UC in the study by Huebner et al. [30]. In this study the investigators put forward an interesting hypothesis that heterozygous carriers of certain *MDR1* variants are more resistant to certain bacterial infections [30]. This hypothesis is based on the data from other diseases, like cystic fibrosis or sickle cell anemia, where heterozygous advantage exists [30]. However, although appealing, this hypothesis still needs to be tested, especially on a functional level.

The G2677T polymorphism is a coding polymorphism resulting in substitution of alanine by serine in the Pgp amino acid sequence. Studies investigating the influence of this polymorphism on Pgp expression have reached conflicting results with Kim et al. reporting association of 2677T allele with higher levels of Pgp expression [14]. However, Kimchi-Sarfaty et al. reported no influence of this polymorphism on expression level and intracellular localisation of Pgp [37]. However, G2677T polymorphism might exert its effect not by influencing levels of expression of Pgp but by altering Pgp stability or affinity for a certain substrate. On the other hand, C3435T has been associated with low levels of Pgp expression in Caucasians [11]. Interestingly, the opposite was found in Japanese subjects emphasizing the interethnical differences in Pgp expression [12]. The association of C3435T with low levels of Pgp expression in caucasians is in concordance with data from animal models with mdr1^−/−^ developing colitis. Interestingly, C3435T is a synonymous SNP not leading to change in amino acid sequence. The possible explanation of the association of a synonymous SNP with altered levels of Pgp expression was offered by finding of moderate reduction of Pgp mRNA stability associated with this SNP [38]. Another possible explanation lies in the high levels of linkage disequilibrium within the *MDR1* gene with C3435T in linkage with the true *causal* variant [5].

In general, our results seem to be in accordance with some of the previously published studies [15,17,20,21,23]. However, in a multitude of studies no association of investigated SNP's and UC was found [16,18,19,22,25,27,29]. The reason for such variability of results in the literature is not clear, however population heterogeneity seems to be the most plausible explanation. In the published studies frequency of the 3435T allele varied from 41%-65.3% in the UC patient group while frequency of 2677T allele varied from 40%-53% [15-30]. Even greater variation of mutant allele frequency can be found in the respective control groups where 3435T allele frequency varied from 37%-63% and 2677T allele frequency varied from 40%-53% [15-30]. The importance of the mutant allele frequency is further emphasized by study by Glas et al. in which the investigators used two separate controls groups. Performing the association analysis with control group 1 that had a 3435T allele frequency of 52.8% no significant association of the 3435T allele and UC was found. However, when control group 2 was used for analysis with 3435T allele frequency of 43.7 a significant association of 3435T allele and UC was found [18]. Due to the fact that the differences in the minor allele frequencies between the IBD group and the control group are quite modest it is quite possible that differences in the minor allele frequencies of the tested SNP's in the controls groups account for the majority of different results in the literature regarding these two SNP's. Importantly, the frequency of 2677T allele in our control group was 35.8% which represents the lowest frequency published to date in a European population. Interestingly, the frequency of 2677T allele, in the ethnically similar, Slovenian control group in the study by Potocnik et al. was also among the lowest reported in the literature (40%) [20]. Our study further emphasizes differences between various European populations and underscores the importance of replication of results of genetic studies in different ethnic groups.

Finally, our study had several weaknesses. IBD patients included in the study represent the IBD population of a tertiary refferal center. As a consequence, majority of patients have extensive disease, especially in the UC group. Therefore, we can not exclude the possibility of bias since our cohort does not include a large number of patients with limited disease of low activity. Furthermore, although we detected a significant association of tested SNP's with UC and CD, the magnitude of the effect is modest. Given our relatively small sample size, our study had only 43% power to detect the effect of this magnitude. Taking this into account, the results of our study, although generally in accordance with previous reports, must be interpreted with caution.

## Conclusion

In conclusion, our results confirm the role of *MDR1* in both CD and especially in UC pathogenesis although the overall influence of *MDR1* variants on disease susceptibility is modest.

## Competing interests

The authors declared that they have no competing interests.

## Authors’ contributions

MB collected and analyzed the data and drafted the manuscript. SCC designed the study, interpreted the data, critically revised the manuscript and approved the final version to be published. NB interpreted the data, drafted the manuscript, critically revised the manuscript and approved the final version to be published. KGR collected the data, critically revised the manuscript and approved the final version to be published. PM collected the data, critically revised the manuscript and approved the final version to be published. AL collected the data, critically revised the manuscript and approved the final version to be published. MC critically revised the manuscript and approved the final version to be published. ZK critically revised the manuscript and approved the final version to be published. BV critically revised the manuscript and approved the final version to be published.

## Pre-publication history

The pre-publication history for this paper can be accessed here:

http://www.biomedcentral.com/1471-230X/13/57/prepub
